# Deep Learning-Based Prediction of Enzyme Optimal pH and Design of Point Mutations to Improve Acid Resistance

**DOI:** 10.1021/acssynbio.5c00679

**Published:** 2025-11-21

**Authors:** Sizhe Qiu, Nan-Kai Wang, Yishun Lu, Jin-Song Gong, Jin-Song Shi, Aidong Yang

**Affiliations:** † Department of Engineering Science, 6396 University of Oxford, Oxford OX1 3PJ, United Kingdom; ‡ Key Laboratory of Carbohydrate Chemistry and Biotechnology, Ministry of Education, School of Life Sciences and Health Engineering, 66374 Jiangnan University, Wuxi 214122, PR China; § Yixing Institute of Food and Biotechnology Co. Ltd, Yixing 214200, PR China; ∥ Oxford E-Research Centre, Department of Engineering Science, University of Oxford, 7 Keble Road, Oxford OX1 3QG, United Kingdom

**Keywords:** enzyme optimal pH, deep learning, sequence-based prediction, self-attention, enzyme engineering, acid resistance

## Abstract

An accurate deep learning predictor of enzyme optimal pH is essential to quantitatively describe how pH influences the enzyme catalytic activity. CatOpt, developed in this study, outperformed existing predictors of enzyme optimal pH (RMSE = 0.833 and *R* ^2^ = 0.479), and could provide good interpretability with informative residue attention weights. The classification of acidophilic and alkaliphilic enzymes and prediction of enzyme optimal pH shifts caused by point mutations showcased the capability of CatOpt as an effective computational tool for identifying enzyme pH preferences. Furthermore, a single point mutation designed with the guidance of CatOpt successfully enhanced the activity of *Pyrococcus horikoshii* diacetylchitobiose deacetylase at low pH (pH = 4.5/5.5) by approximately 7%, suggesting that CatOpt is a promising *in silico* enzyme design tool for pH-dependent enzyme activities.

## Graphical abstract

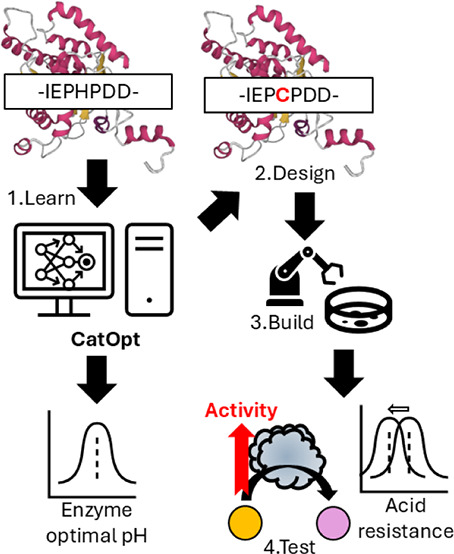

## 1 Introduction

In the era of synthetic biology, enzymes play a crucial role in industrial processes, such as food fermentation, waste transformation, and eco-friendly bio-manufacturing of chemical products.^1^ In those industrial processes, pH is an important influencing factor of enzyme catalytic activity, as the increase or decrease of pH can affect enzyme protein conformations.^2–4^ Each enzyme has an optimal pH (pH_opt_) where its maximum catalytic rate is attained. Therefore, an accurate enzyme pH_opt_ predictor is highly desirable for enzyme mining and engineering, enabling the discovery of enzymes suited to specific environmental pH and supporting the efforts to enhance catalytic activity within targeted pH ranges.

To fill the knowledge gap of enzyme pH_opt_ in enzyme databases (e.g., BRENDA,^5^ uniprot^6^) caused by the high cost of enzyme assays,^7^ several machine learning models were developed to make predictions from protein sequences, but most of them could only predict pH_opt_ ranges (acidophilic or alkaliphilic).^8–11^ MeTarEnz^12^ used random forest regression to quantitatively predict pH_opt_ values, but the accuracy was low (MSE = 1.648 and *R* ^2^ = 0.195). Recently, the use of protein language models improved the prediction accuracy of pH_opt_. EpHod^13^ and Seq2pHopt^14^ achieved RMSE scores close to 1 using ESM-1 ^15^ and ESM-2,^16^ respectively. Subsequently, OphPred^17^ surpassed EpHod and Seq2pHopt using ESM-2 and XGBoost,^18^ but it lacked interpretability for protein residues. Besides, none of the existing models has been applied to enzyme engineering.

With the aim to build a predictor of enzyme pH_opt_ with good accuracy and interpretability, this study constructed a deep learning model, named CatOpt, with a pre-trained language model of proteins, multi-scale convolutional neural network (CNN), multi-head self-attention, and residual dense neural networks. CatOpt allows the interpretation of residue attention weights, which helps to decipher the key sequence information for enzyme pH_opt_. Case studies on classifying acidophilic and alkaliphilic enzymes and predicting enzyme pH_opt_ changes by point mutations were carried out to examine CatOpt’s performance on *in silico* enzyme selection. The predictor-guided engineering of*Pyrococcus horikoshii*diacetylchitobiose deacetylase to enhance acid resistance, suited to its acidic working environment,^19^ demonstrated that CatOpt can function as a useful computational tool for enzyme engineering.

## 2 Methods

### 2.1 Construction of the Deep Learning Model

The training and test datasets of optimal pH (pH_opt_) were obtained from the Zenodo repository of EpHod.^13^ The training set in this study was merged with the original training and validation sets of EpHod. Using the same training and test datasets as EpHod avoided data leakage in model comparison (Section 3.1). Sequence identity scores between all protein sequences in the test and training datasets were calculated using MMseqs2 ^20^ for subsequent model evaluation. The model architecture of CatOpt consisted of protein sequence embedding by ESM-2,^16^ multi-scale convolutional neural network (CNN), multi-head self-attention network, and residual dense blocks (Figure 1). As ESM-2 had good performance in Seq2pHopt and OphPred, it was chosen to generate protein sequence embeddings in this work.^13,17^

**Figure 1:**
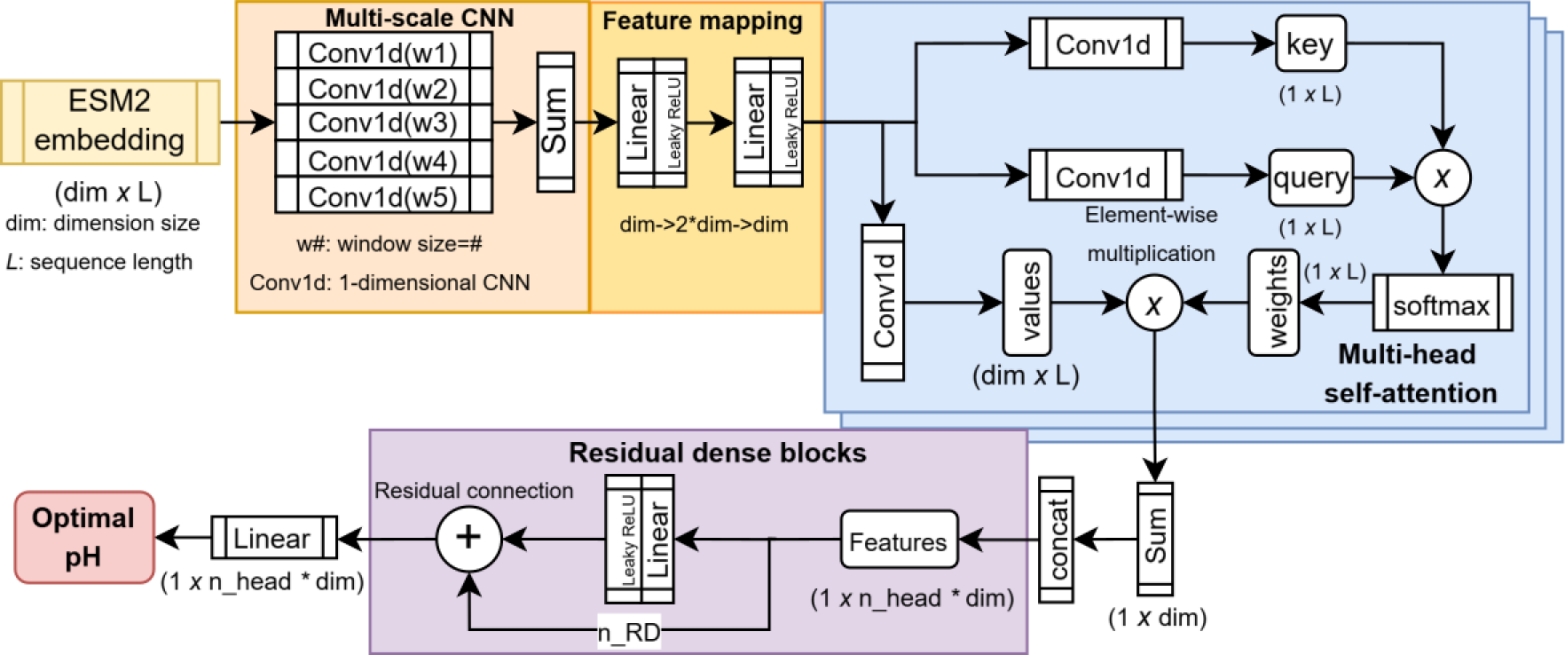
Model architecture of CatOpt. L: protein sequence length; dim: embedding dimension size; Conv1d: 1-D convolutional layer; ⊗: element-wise multiplication; *n*_head: the number of heads in multi-head attention; concat: concatenation; RD: residual dense block, a dense layer with residual connection.

First, the protein sequence embeddings (*r* ∈ *R^dim^* ^×*L*^, L: sequence length, dim = 320) were computed using the esm2_t6_8M_UR50D model.^16^ The embeddings (*r*) were first passed to the multi-scale CNN to extract features with sliding window sizes of 1, 2, 3, 4, and 5. The outputs from CNNs with different sliding window sizes were added in an element-wise way. Then, the outputs of the multi-scale CNN were passed to a 2-layer linear featuremap. As alternatives to the multi-scale CNN, CNNs with fixed sliding window sizes were also tested (see SI, Figure S3). In linear feature mapping, the feature dimension was transformed from dim to 2*dim, and then back to dim.

In multi-head self-attention, the outputs of the 2-layer linear feature map were transformed to values (*v^i^* ∈ *R^dim^* ^×*L*^, *i*: attention head index), keys (*k^i^* ∈ *R* ^1×*L*^) and queries (*q^i^* ∈ *R* ^1×*L*^) via 1-D CNNs. The self-attention weights (*w^i^* ∈ *R* ^1×^ *^L^*) were computed with element-wise multiplication of keys and queries and a softmax function (eq 1). Next, the element-wise products of values and weights were computed and summed at the dimension of sequence length. The weighted features (*x^i^* ∈ *R^dim^*) from all attention heads were concatenated as the inputs (*x_concat_* ∈ *R^n^* ^_^ *^head×dim^*, *n*_head: number of attention heads) for residual dense blocks. Each residual dense block consisted of a linear layer, Leaky ReLU,^21^ and a residual addition operator, ⊕. In the end, a linear layer used the outputs from residual dense blocks to regress for enzyme pH_opt_ values.

1 $$w^{i}=\mathrm{softmax}\left(q^{i}\times k^{i}\right),\;\forall \mathrm{attention}\;\mathrm{head}\;i=1-n\text{\_}\mathrm{head}$$

### 2.2 Deep Learning Model Training

For the training process, batch training was used (batch size = 32) for the efficiency and generalizability of the deep learning neural network. Adam optimization algorithm^22^ was used to update neural network weights iteratively. The loss function was mean squared error (MSE). The initial learning rate was 0.0005, and the learning rate decayed by 50% for every 10 epochs to prevent overfitting. Before model training started, 10% of the training set was split out as the validation set, and target values were rescaled as $\frac{pH_{opt}}{14}$ . During the training process, the prediction accuracy of the model was evaluated with root mean squared error (RMSE), mean average error (MAE), and r-squared (*R* ^2^) (see SI, eq S1-3). For details of software and hardware, please see the Section S1.1 of the Supplementary Information. There were 2 hyperparameters in CatOpt, number of attention heads (*n*_head) and number of residual dense blocks (*n*_RD), and hyperparameter optimization was performed on *n*_head = 4, 5, 6 and *n*_RD = 3, 4, 5 (see SI, Figure S2).

### 2.3 Interpretation of Residue Attention Weights

To investigate how enzyme pH_opt_ was predicted from the amino acid sequence, the average residue attention weights (*w_avg_* ∈ *R* ^1*^ *^L^*) were computed by averaging the weights across all attention heads (eq 2). Then, the average residue attention weights were mapped to the protein sequence, together with annotated acidic/basic residues, active and binding sites obtained from the uniprot database.^6^ The spatial distribution of residue attention weights and annotated protein sequence features could assist in revealing the key sequence information influencing the enzyme pH_opt_.

2 $$w_{\mathrm{avg}}=\overset{n\text{\_}\mathrm{head}}{\underset{i=1}{\sum }}w^{i}/n\text{\_}\mathrm{head},,i:\mathrm{attention}\;\mathrm{head}\;\mathrm{index}$$

### 2.4 Predictor-Guided Design of Single Point Mutations

CatOpt was used to design single point mutations to enhance the acid resistance of*Pyrococcus horikoshii* diacetylchitobiose deacetylase (PhDac), an enzyme catalyzing the production of glucosamine (GlcN) from N-acetylglucosamine (GlcNAc).^19^ Because the fermentation environment of diacetylchitobiose deacetylase is usually acidic, it is desirable to enhance its enzyme activity under low pH.^23^ For 3 × 21 sites centered at three substrate binding sites (D46, R92, and H152, −10 site to +10 site), all possible amino acid substitutions were considered, and thus there were totally 1197 mutated sequences. For all mutants, pH_opt_ and turnover numbers (*k_cat_*) were predicted by CatOpt and DLTKcat,^24^ respectively. DLTKcat was used to predict turnover numbers of mutants and filter out mutants with low catalytic efficiency. Designed point mutations were selected with an arbitrary threshold of predicted pH_opt_ < 7. In this study, site directed mutagenesis, protein expression and purification of PhDac mutants followed the same procedure as in Huang et al., 2021.^19^

### 2.5 Measurement of Enzyme Activity

The enzyme activity measurement method was adapted from Jiang et al., 2019 ^25^ with several modifications. In summary, the reaction solution comprised 1 mL of citrate buffer (50 mM, pH 4.5 to 5.5), 50 g/L of GlcNAc, and 100 μL crude enzyme (i.e., designed mutants). The reaction was performed in a metal bath at 40 °C and 900 rpm for 20 min. Subsequently, the reaction was halted by the addition of 50 μL of 0.5 M HCl. The mixture was then centrifuged at 12,000 rpm for 5 min. After centrifugation, 10 μL of the supernatant was combined with 100 μL of the OPA detection reagent (composed of 5 mg OPA, 10 μL of 1.0 M dithiothreitol, and 100 μL of alcohol in 10 mL of sodium carbonate buffer), and the absorbance was measured at 330 nm using the Infinite M200 PRO Spectrum spectrophotometer (Tecan Trading AG; Switzerland). One unit of enzyme activity was defined as the amount of the enzyme liberating 1 μM GlcN in 1 h at 40 °C.

## 3 Results

### 3.1 CatOpt Outperformed Existing Predictive Models

First, the hyperparameter optimization on the number of attention heads (*n*_head) and residual dense blocks (*n*_RD) found that *n*_head = 4 and *n*_RD = 4 is the best set of hyperparameters in the search scope (see SI, Figure S2). Also, the performance comparison of CNNs with fixed sliding window sizes (3, 4, 5) and the multi-scale CNN of 5 different window sizes justified the use of multi-scale CNN in CatOpt (see SI, Figure S3). Then, CatOpt, with the optimal set of hyperparameters, achieved a prediction accuracy of *R* ^2^ = 0.479, MAE = 0.607 and RMSE = 0.833 on the hold-out test set (Figure 2AB and see SI, Figure S4). In model comparison on the same test set provided by the Zenodo repository of EpHod,^13^ CatOpt outperformed Seq2pHopt, EpHod, and OphPred (Figure 2C). With respect to prediction errors at *pH*_opt_ < 6, CatOpt had a RMSE of 1.189, close to EpHod (RMSE = 1.180) and lower than OphPred (RMSE = 1.393) and Seq2pHopt (RMSE = 1.375) (Figure 2D). At pH_opt_ > 8, CatOpt had a RMSE of 1.319, slightly lower than EpHod (RMSE = 1.339) and OphPred (RMSE = 1.331) (Figure 2E). For 6 enzyme classes (EC1-6), CatOpt had lower RMSEs than the other 3 models in oxidoreductases (EC1), hydrolases (EC3), lyases (EC4), and isomerases (EC5), and close RMSEs to EpHod and OphPred in transferases (EC2) and ligases (EC6) (Figure 2F). There were only 22 translocases (EC7) in the test set (see SI, Figure S1), much fewer than the other enzyme classes, therefore EC7 was not included in model performance evaluation. For enzymes with varying identity scores to those in the training dataset, CatOpt exhibited lower RMSEs than the other 3 models in <30% and >60% identity score ranges, and a comparable RMSE to OphPred in the 30–60% range (Figure 2G). In general, CatOpt exhibited good accuracy and outperformed existing predictive models of enzyme pH_opt_.

**Figure 2:**
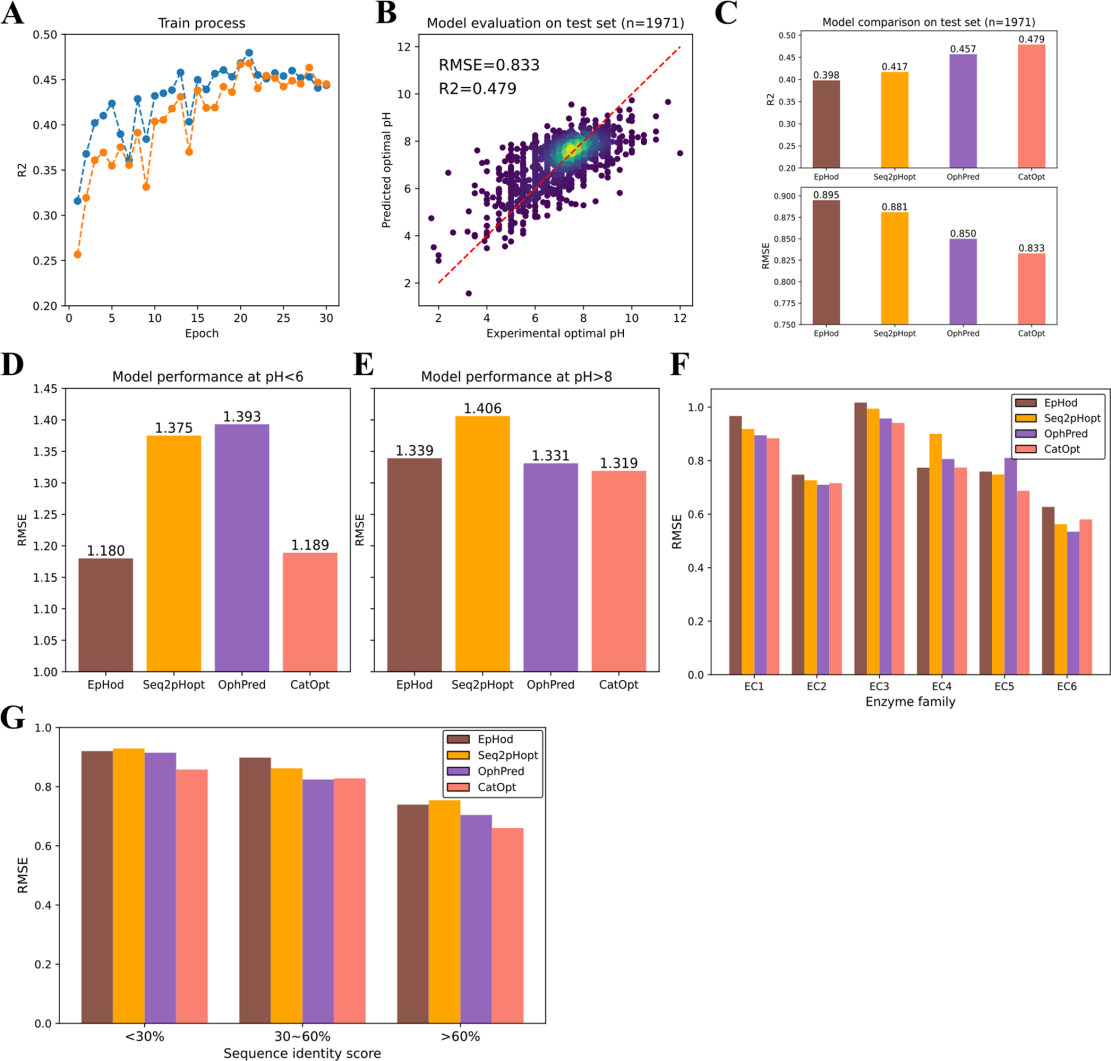
Model performance evaluation of CatOpt. (A) The *R* ^2^ scores of pH_opt_ prediction during the training process. Blue dotted curve: test set; Orange dotted curve: dev set (validation set). (B) Experimental and predicted pH_opt_ by CatOpt (RMSE = 0.833 and *R* ^2^ = 0.479). (C) Prediction accuracy comparison of EpHod (*R* ^2^ = 0.399, RMSE = 0.895), Seq2pHopt (*R* ^2^ = 0.417, RMSE = 0.881), OphPred (*R* ^2^ = 0.457, RMSE = 0.85), and CatOpt (*R* ^2^ = 0.479, RMSE = 0.833) on the same test set. (D) Prediction accuracy comparison of 4 models at low (pH_opt_ < 6) value range. (E) Prediction accuracy comparison of 4 models at high (pH_opt_ > 8) value range. (F) Prediction accuracy comparison of EpHod, Seq2pHopt, OphPred, and CatOpt for different enzyme classes (EC 1–6). (G) Prediction accuracy comparison of EpHod, Seq2pHopt, OphPred, and CatOpt for different sequence identity score ranges (<30%, 30–60%, >60%).

### 3.2 Identifying the pH Preferences of Enzymes and Microorganisms

After the prediction accuracy of CatOpt had been validated (Section 3.1), this study proceeded to examine its ability to identify the pH preferences of enzymes and microorganisms. The benchmark dataset of AcalPred (54 acidophilic enzymes and 68 alkaliphilic enzymes)^8^ was used in the identification of acidophilic and alkaliphilic enzymes. Notably, this dataset had no overlap with the training dataset of CatOpt. The predicted pH_opt_ values of alkaliphilic enzymes were significantly higher than those of acidophilic enzymes (Figure 3A). With the cutoff of pH_opt_ = 7.0, CatOpt classified acidophilic and alkaliphilic enzymes with an accuracy of 91.8%, 50 acidophilic enzymes and 62 alkaliphilic enzymes were accurately identified (Figure 3BC). In 10 misclassified enzymes, CatOpt mislabelled O60502, P07311, P25026, Q9ET64 as alkaliphilic enzymes, and A7ZQK5, P04055, Q08D86, Q16853, Q59516, Q9Y5Z0 as acidophilic enzymes. EpHod performed slightly worse than CatOpt, misclassifying 13 enzymes (see SI, Figure S9). The host organisms of misclassified enzymes were mainly neutrophilic, such as*Homo sapiens*and*Pseudomonas pyrrocinia*. This is likely because enzymes from neutrophiles typically have near-neutral pH_opt_ values, making it difficult to classify them as either acidophilic or alkaliphilic. Overall, this case study demonstrated that CatOpt could discriminate enzymes with different pH preferences.

**Figure 3:**
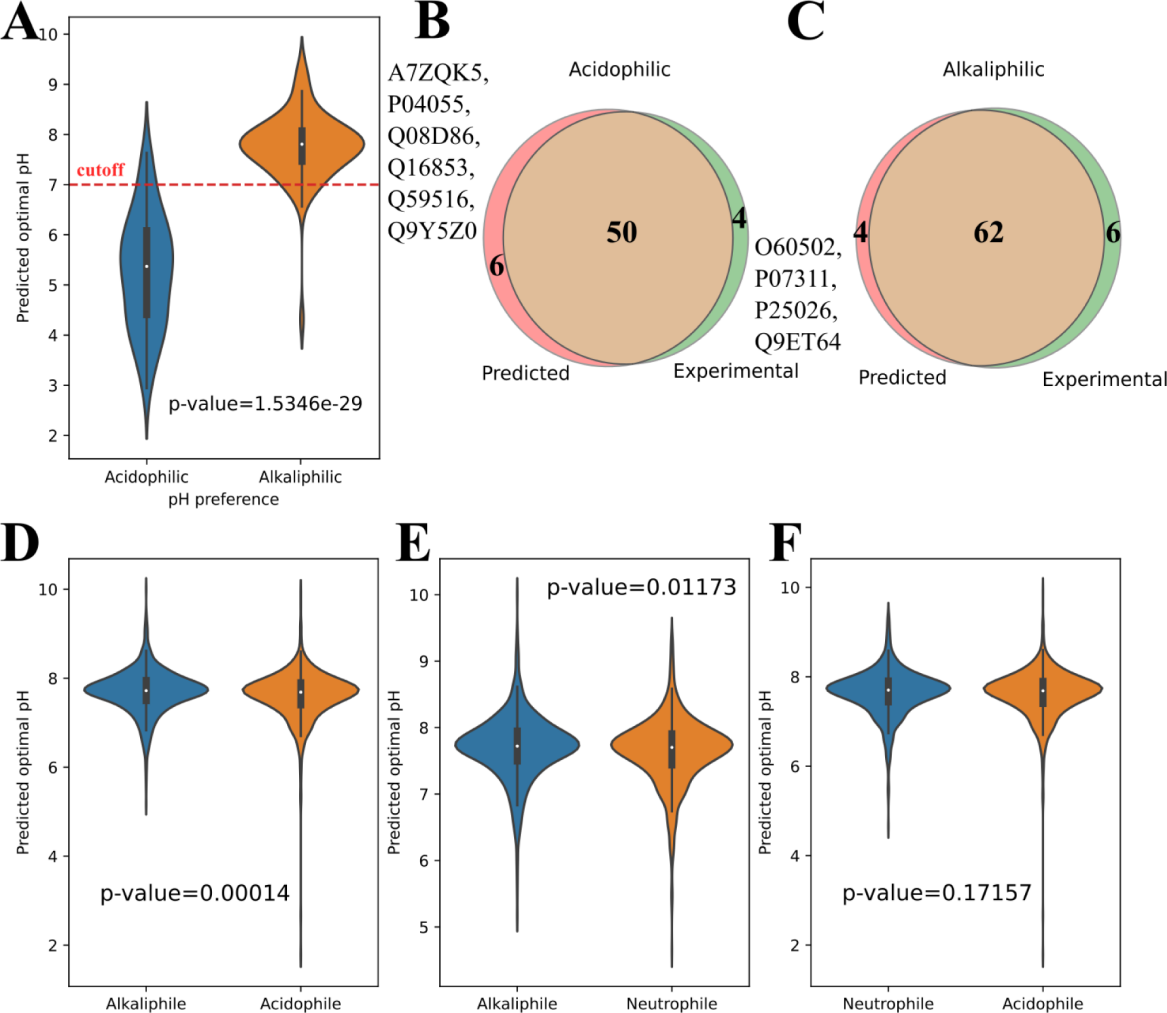
Performance of CatOpt on the pH preference of enzymes and microorganisms. (A) The distribution of predicted pH_opt_ values of 54 acidophilic enzymes and 68 alkaliphilic enzymes (*p*-value < 0.001). (B) The Venn diagram of predicted and experimental acidophilic enzymes. (C) The Venn diagram of predicted and experimental alkaliphilic enzymes. (D) The distribution of predicted pH_opt_ values of enzymes from alkaliphilic and acidophilic microorganisms (*p*-value < 0.001). (E) The distribution of predicted pH_opt_ values of enzymes from alkaliphilic and neutrophilic microorganisms (*p*-value < 0.05). (F) The distribution of predicted pH_opt_ values of enzymes from neutrophilic and acidophilic microorganisms (*p*-value > 0.05).

Next, pH_opt_ values were predicted for catalytic enzymes belonging to 3 acidophilic, 3 neutrophilic, and 3 alkaliphilic microorganisms (see SI, Figure S5). Enzyme protein sequences were all obtained from the uniprot database.^6^ CatOpt could discriminate alkaliphilic and acidophilic microorganisms with significantly different distributions of predicted enzyme pH_opt_ values, the same for alkaliphilic and neutrophilic microorganisms (Figure 3D,E). However, there was no significant difference between predicted pH_opt_ values of enzymes from acidophilic and neutrophilic microorganisms (Figure 3F). Despite that CatOpt could identify the pH preferences of enzymes, it could not accurately classify acidophilic, neutrophilic, and alkaliphilic microorganisms based on distributions of predicted enzyme pH_opt_ values.

### 3.3 Residue Attention Weights Capture Key Sequence Information

To investigate how residue attention weights capture important sequence information, this study compared attention weights on different types of residues across acidophilic, neutrophilic, and alkaliphilic enzymes in the hold-out test set. For both acidic and basic residues, the attention weights of acidophilic and neutrophilic enzymes were significantly higher than the weights of acidophilic enzymes (Figure 4A), which revealed the importance of ionizable residues on enzyme pH_opt_. In all three types of enzymes, the attention weights on nonpolar residues were significantly higher than the weights on polar residues (Figure 4B). Previous studies have shown that ionizable and polar residues are associated with pH-dependent transient states of enzymes, where catalysis occurs.^26–28^ Therefore, the significantly different distributions of attention weights on ionizable, polar, and other residues indicated that CatOpt effectively identified key residues related to pH-dependent enzyme catalytic activity.

**Figure 4:**
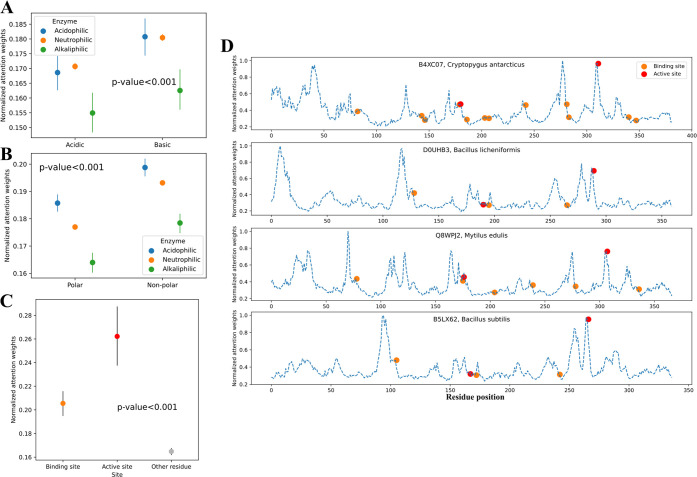
Analysis of residue attention weights. (A) The attention weights of acidic and basic residues of acidophilic, neutrophilic, and alkaliphilic enzymes. (B) The attention weights of polar and non-polar residues of acidophilic, neutrophilic, and alkaliphilic enzymes. (C) The attention weights on binding sites, active sites and other residues. (D) Representative examples of residue attention weights, positions of binding and active sites of 4 mannan endo-1,4-beta-mannosidases (EC:3.2.1.78). The uniprot IDs are B4XC07, D0UHB3, Q8WPJ2, and B5LX62. Blue dashed curve: normalized attention weights; orange dot: binding site; red dot: active site.

Next, 173 enzymes with annotated active and binding sites were selected from the test set. The active sites are regions where chemical reactions happen, and the binding sites are residues where substrates bind. The attention weights on active and binding sites were significantly higher than attention weights on other residues (Figure 4C), suggesting that residue attention weights could capture important residues for enzyme catalysis. For example, the active sites of 4 mannan endo-1,4-beta-mannosidases were mostly close to peaks of residue attention weights, although the weights were not indicative for binding sites (Figure 4D). In presented 4 enzymes, there were high attention weight peaks that did not correspond to annotated active and binding sites. Unfortunately, these high weight peaks could not be interpreted with existing sequence annotations. In a nutshell, residue attention weights in CatOpt provided good interpretability by capturing key sequence information, i.e., ionizable residues, active and binding sites.

### 3.4 Prediction of Optimal pH Shifts Caused by Point Mutations

To examine the inference ability of CatOpt on how point mutations affect enzyme pH_opt_, this study used it to predict pH_opt_ values of wild-types (WTs) and mutants for*Pyrococcus horikoshii*diacetylchitobiose deacetylase (PhDac),^19^ *Bacillus circulans*xylanase (BCX),^29^ *Lactiplantibacillus plantarum*tannase (TanBlp),^30^ and*Clonostachys rosea* zearalenone hydrolase (CRZHD)^31^ (see SI, Table S1). The experimental data of those enzymes was not included in the training set of CatOpt. The overall prediction error for WTs and mutants of those 4 different enzymes was RMSE = 1.35, MAE = 1.12, and R^2^ = 0.298 (Figure 5A).

**Figure 5:**
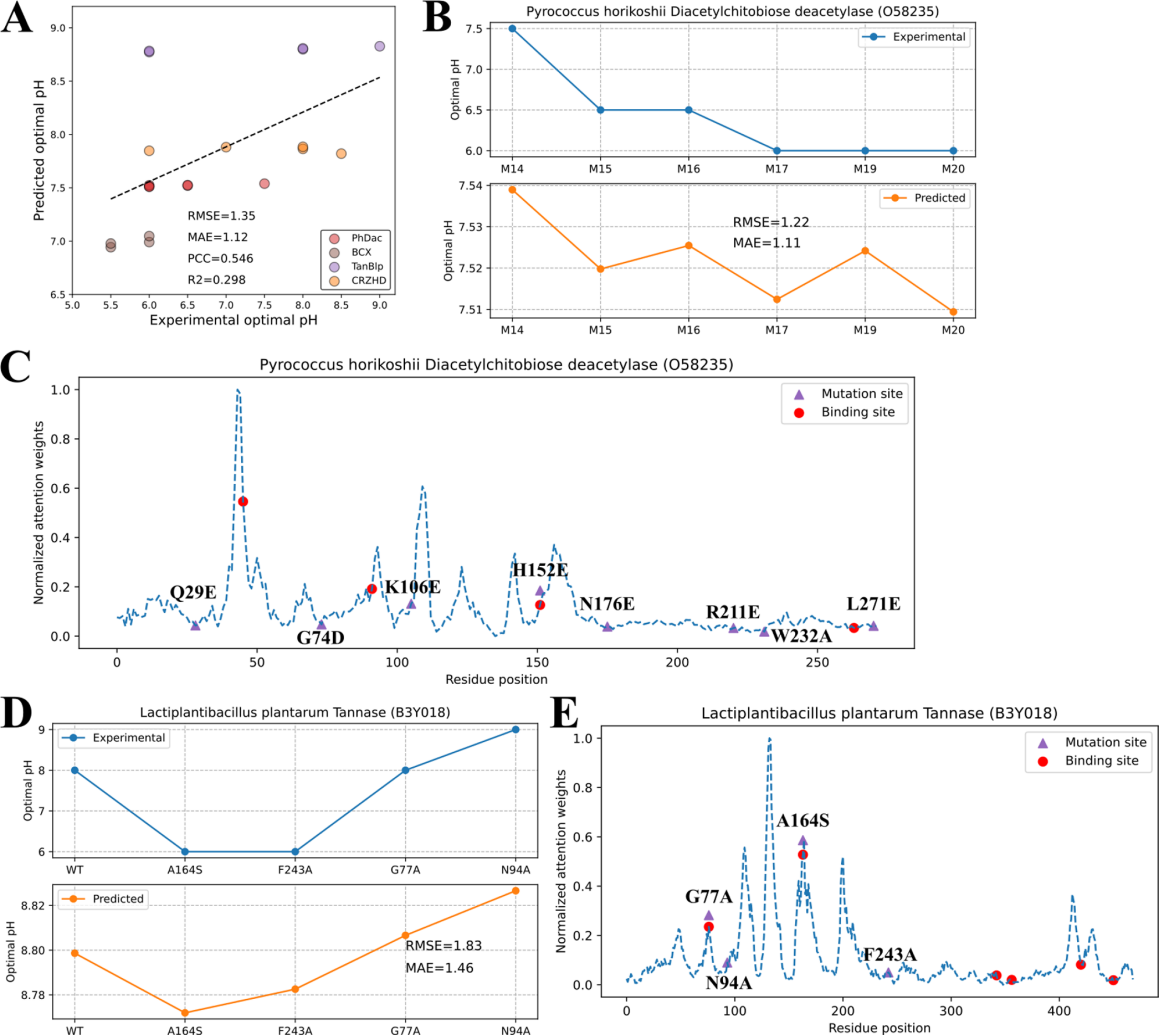
Prediction of enzyme pH_opt_ shifts caused by mutations. (A) Experimental and predicted enzyme pH_opt_ of WTs and mutants of 4 different enzymes (RMSE = 1.35, MAE = 1.12, PCC = 0.546, *R* ^2^ = 0.298). PCC: Pearson correlation coefficient, PhDac:*Pyrococcus horikoshii*diacetylchitobiose deacetylase, BCX:*Bacillus circulans* xylanase, TanBlp:*Lactiplantibacillus plantarum*tannase, CRZHD:*Clonostachys rosea*zearalenone hydrolase, WT: wild-type. (B) Experimental and predicted enzyme pH_opt_ of 6 mutants of PhDac. (C) Residue attention weights of PhDac and positions of substrate binding sites and point mutations in 6 mutants. (D) Experimental and predicted enzyme pH_opt_ of WT and mutants of TanBlp. (E) Residue attention weights of TanBlp and positions of substrate binding sites and point mutations in 4 mutants.

For PhDac, the prediction by CatOpt qualitatively accounted for the downshift of pH_opt_ in M15 and M16 in comparison to M14, and in M17 and M20 in comparison to M15 and M16, but the numerical difference of predicted pH_opt_ among 6 mutants was small (Figure 5B). The residue attention weights of PhDac captured substrate binding sites with high peaks (e.g., D46) (Figure 5C), although the spatial distribution of weights could not explain effective point mutations causing down-shift of pH_opt_ (e.g., Q29E). The highest peak of attention weights was at residue 44, suggesting a potential effective site for point mutations. For TanBlp, CatOpt quantitatively predicted the downshift of pH_opt_ caused by A164S and F243A in comparison to WT, and the upshift of pH_opt_ by G77A and N94A in comparison to A164S and F243A (Figure 5D). Two substrate binding sites of TanBlp (G77 and A164) and two effective point mutations (G77A and A164S) were identified by high peaks of residue attention weights (Figure 5E). BCX and CRZHD were not included in further analysis (see SI, Figure S7), due to lack of protein sequence annotation. Generally speaking, this case study demonstrated CatOpt’s capability to predict the effect of point mutations on enzyme pH_opt_, although the numerical differences of predicted enzyme pH_opt_ for WTs and mutants were smaller than those observed in experimental measurements.

### 3.5 Predictor-Guided Engineering of Diacetylchitobiose Deacetylase to Enhance Acid Resistance

CatOpt was used as a computational design tool to enhance the acid resistance of PhDac (Figure 5BC), which is used in the environmentally-friendly manufacturing of GlcN.^19^ Enzyme pH_opt_ and turnover number values of 1197 mutants with single point mutations based on M20 (see SI, Table S1) were predicted (Figure 6A). 5 mutants with lowest predicted pH_opt_ values, which also had relatively high turnover number values, were selected to examine their activities at pH = 4.5 and 5.5. Compared to M20, H44C improved the activities of PhDac at pH = 4.5 and 5.5 by around 7% (*p*-value < 0.05), H44D improved the activity at pH = 4.5 by around 2% (nonsignificant) (Figure 6B). The other 3 selected mutants (M20 + H44I, M20 + H44N, M20 + H44P) did not enhance the acid resistance, but their activities at pH = 4.5 were all higher than activities at pH = 5.5 (Figure 6B), which suggested the down-shift of pH_opt_. Both effective point mutations (H44C and H44D) substituted a basic residue (histidine (H)) with an acidic residue (cysteine (C) and aspartate (D)), possibly stabilizing the transition state at the substrate binding site, D46, under acidic pH conditions.^32^ However, the stabilizing effect of acidic residues close to PhDac’s substrate binding sites remained a hypothesis, pending future experimental validation. In addition, enzyme activities of 5 un-selected mutants (Figure 6A) were also measured, and they showed weaker acid resistance than M20 (Tables S2, S3, Figure S8). In short, the success of computationally designed point mutations showed the usefulness of CatOpt as a design tool of enzyme engineering.

**Figure 6:**
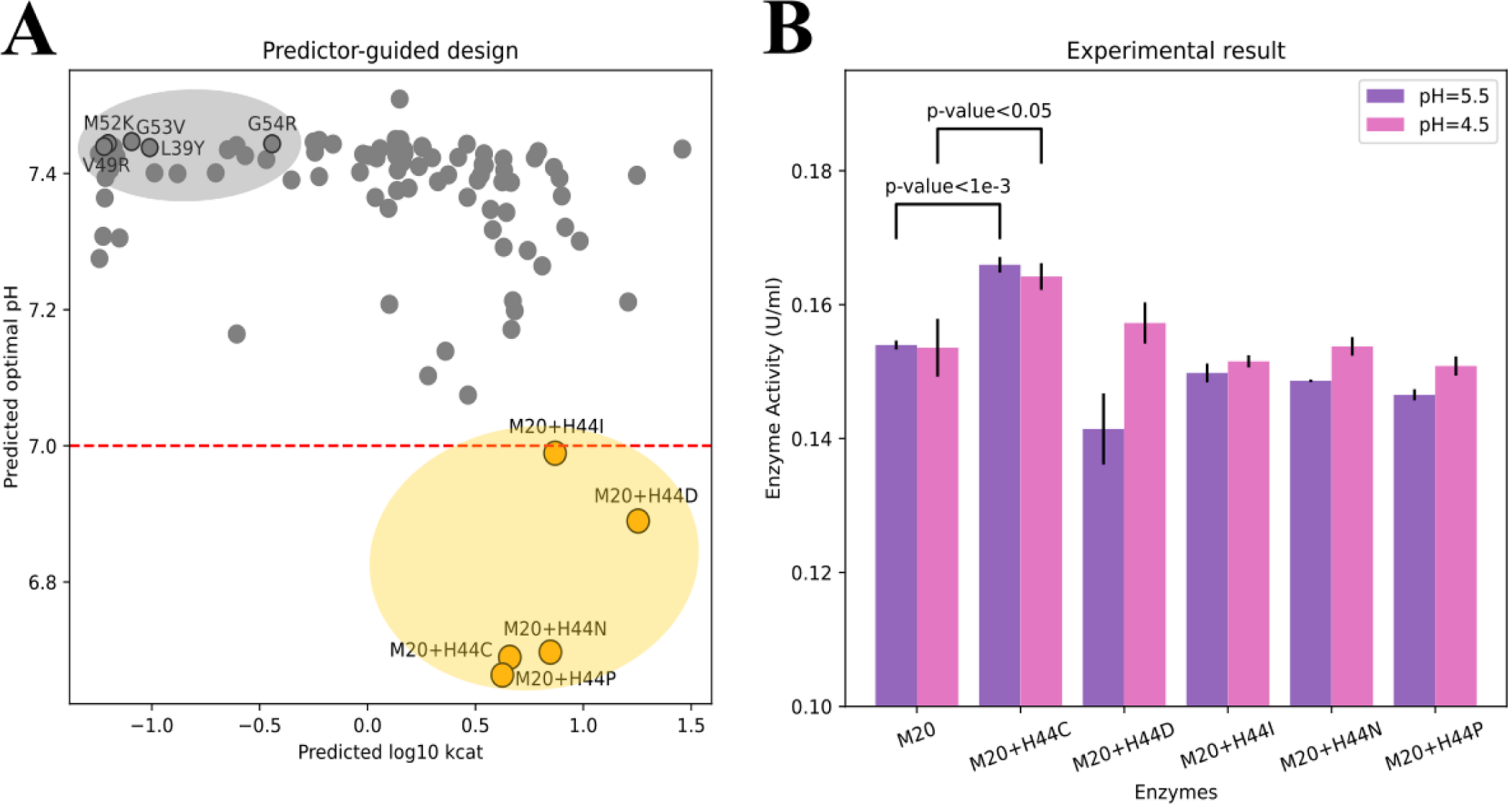
Predictor-guided engineering of*Pyrococcus horikoshii*diacetylchitobiose deacetylase (PhDac). (A) Predicted enzyme pH_opt_ and turnover number values (log_10_ *k*_cat_) of mutants with single point mutations based on M20 (only lower 10% of predicted pH_opt_ values were presented). Orange dots represent 5 selected mutants: M20 + H44I, M20 + H44D, M20 + H44N, M20 + H44C, M20 + H44P. Grey dots represent the remaining un-selected mutants, grey dots with black edges represent M20 + G53 V, M20 + M52K, M20 + G54R, M20 + V49R, M20 + L39Y. (B) Enzyme activities (U/mL) at pH = 4.5 and 5.5 for M20 and 5 selected mutants. All measurements have 3 replicates (see SI Table S2, S3). The error bars represent standard deviations.

## 4 Discussion

To address the limitations of existing predictive models of pH_opt_ (e.g., low accuracy or lack of interpretability), this study developed CatOpt, a deep learning model capable of predicting enzyme pH_opt_ directly from protein sequences. Main components of CatOpt were ESM-2 embedding generation, multi-scale CNN, multi-head self-attention, and residual dense neural networks (Figure 1). Compared with one-hot encoding^33^ or k-mer based dictionary embedding,^24,34^ ESM-2, as a pre-trained large language model of proteins, can transfer the knowledge of protein structures and functions from a large dataset of millions of protein sequences to this prediction task using thousands of protein sequences.^16^ The advantage of multi-scale CNN with different window sizes over CNN with a fixed window size lies in ensembling information to enrich the representation of protein sequence features.^35^ In contrast to multi-head light attention in Seq2Topt/Seq2pHopt,^14^ self-attention in CatOpt can model dependencies between different regions of the protein sequence.^36^ Additionally, the use of residue dense neural networks instead of multiple linear layers could effectively reduce the vanishing and exploding gradient issues in deep neural networks.^37^ Consequently, CatOpt outperformed existing enzyme pH_opt_ predictors (e.g., OphPred) with RMSE = 0.833 and *R* ^2^ = 0.479, and provided good interpretability with informative residue attention weights (Section 3.3).

The classification of acidophilic/alkaliphilic enzymes (Section 3.2), prediction of enzyme pH_opt_ shifts by point mutations (Section 3.4), and predictor-guided engineering of PhDac (Section 3.5) demonstrated that CatOpt could be applied to enzyme mining and computational design of enzymes via fast screening the effect of point mutations. Besides *in silico* screening, the combination of generative deep learning and CatOpt might lead to automatic generation of novel acidophilic or alkaliphilic enzymes through predictor-guided generator optimization.^38^ Moreover, like EpHod^13^ and Seq2Topt,^14^ CatOpt can also be trained to predict multiple protein properties with a single sequence-based model. Besides, the informative attention weighted protein features extracted by CatOpt (Section 3.3) could be used in other prediction tasks of enzyme catalytic activity, such as the prediction of pH-dependent enzyme turnover numbers,^39,40^ via transfer learning. Therefore, beyond predicting a specific protein property, CatOpt holds the potential to serve as a protein foundation model^41^ that can be adapted for diverse downstream tasks in protein science.

Despite the achievement of CatOpt outlined above, some limitations still exist and hinder its performance. Similar to Seq2Topt/Seq2pHopt,^14^ the accuracy of CatOpt was also affected by the imbalance of the training dataset. Oversampling and loss reweighting can mitigate the imbalance, but the prediction accuracy at low and high value ranges will still be relatively low.^13,14^ Using high-throughput enzyme assays to append entries at the ranges of pH_opt_ < 6 and pHopt > 8 to the dataset is necessary to further improve the performance of pH_opt_ prediction. The neglect of environmental factors influencing enzyme pH_opt_ is another shortcoming, such as temperature^42^ and ionic strength.^43^ These environmental factors can affect the protein conformation and thereby complicate the relationship between pH and enzyme catalytic activity. The inclusion of enzyme assay metadata could help account for environmental factors and improve the prediction accuracy of enzyme pH_opt_, although a large portion of enzyme assay results curated from databases currently lack such information. In the attempt to identify organismal pH preferences (Section 3.2), CatOpt failed to differentiate neutrophilic and acidophilic microorganisms with distributions of predicted enzyme pH_opt_ values. The distribution of experimental enzyme pH_opt_ values indicates that most enzymes in acidophilic microorganisms have pH_opt_ in the range of 6–8, just like neutrophilic microorganisms (see SI, Figure S6). Therefore, the relationship between microbial growth pH_opt_ and enzyme pH_opt_ still remains to be investigated.

In conclusion, CatOpt is an interpretable deep learning predictor of enzyme pH_opt_ that demonstrated improved accuracy compared to existing tools, despite the limitations discussed above. As envisaged, CatOpt can potentially accelerate enzyme discovery for desired properties from “biological dark matter” and enzyme engineering with *in silico* design.

## Supplementary Material



## Data Availability

The code and data are openly available at https://github.com/SizheQiu/CatOpt and Supporting Information.

## Notes

Supplementary Methods: S1.1 Software and code availability. Supplementary Methods: S1.2 Evaluation metrics. Supplementary Figures: Figure S1. Distributions of enzyme optimal pH values in the training set (7884 entries) and test set (1971 entries), and distributions of enzymes from different EC families in both sets. Supplementary Figures: Figure S2. Hyperparameter optimization of enzyme optimal pH prediction for number of attention heads (*n*_head) and residual dense blocks (*n*_RD). *n*_RD = 4, *n*_head = 4 was chosen as the best set of hyperparameters. Supplementary Figures: Figure S3. Performance comparison of CNNs with fixed sliding window sizes (3, 4, 5) and multi-scale CNN used in CatOpt. *n*_RD = 4, *n*_head = 4. Supplementary Figures: Figure S4. The RMSE, MAE, *R* ^2^ scores of CatOpt during the training process. Supplementary Figures: Figure S5. Predicted optimal pH values for enzymes in 9 microorganisms. Acidophiles: *Acidithiobacillus ferrooxidans*, *Lactobacillus acidophilus*, and *Thermoplasma acidophilum*; Neutrophiles: *Bacillus subtilis*, *Salmonella enterica*, and *Staphylococcus aureus*; Alkaliphiles: *Clostridium paradoxum*, *Alkalihalobacillus alcalophilus*, and *Natronomonas pharaonis*. Supplementary Figures: Figure S6. Experimental optimal pH values for enzymes in 3 acidophilic microorganisms and 2 neutrophilic microorganisms. Supplementary Figures: Figure S7. (A) Experimental and predicted enzyme pH_opt_ of wild-types and mutants of BCX. (B) Experimental and predicted enzyme pH_opt_ of wild-types and mutants of CRZHD. Supplementary Figures: Figure S8. Enzyme activities (U/mL) at pH = 4.5 and 5.5 for M20 and M20 + G53V, M20 + M52K, M20 + G54R, M20 + V49R, M20 + L39Y. Supplementary Figures: Figure S9. The performance of EpHod on the pH preference of enzymes in the AcalPred dataset. Supplementary Tables : Table S1. Information of wild-type and mutated enzymes used in case studies. Supplementary Tables : Table S2. The measured enzyme activities (U/mL) of PhDac mutants at pH = 5.5. Supplementary Tables : Table S3. The measured enzyme activities (U/mL) of PhDac mutants at pH = 4.5 (PDF)

## Abbreviations

BCX: *Bacillus circulans*xylanase
CNN: convolutional neural network
CRZHD: *Clonostachys rosea*zearalenone hydrolase
GlcN: glucosamine
GlcNAc: N-acetylglucosamine
Leaky ReLU: leaky rectified linear unit
MAE: mean absolute error
MSE: mean squared error
PhDac: *Pyrococcus horikoshii*diacetylchitobiose deacetylase
pH_opt_: optimal pH
RD: residual dense block
*R* ^2^: r-squared, the coefficient of determination
RMSE: root mean squared error
TanBlp: *Lactiplantibacillus plantarum*tannase
WT: wild-type
