# Supplementary material for: Deep Learning-Based Prediction of Enzyme Optimal pH and Design of Point Mutations to Improve Acid Resistance
Source: ACS Synth Biol. 2025 Nov 21;14(12):4897–906. doi: 10.1021/acssynbio.5c00679 (PMC12723737; doi:10.1021/acssynbio.5c00679)
Supplement: Supplementary file 1 [file sb5c00679_si_001.pdf]

Deep learning-based prediction of enzyme optimal pH and design of point mutations to improve acid resistance

Authorship

Sizhe Qiu[1‡], Nan-Kai Wang[2,3‡], Yishun Lu[1,4‡], Jin-Song Gong[2,3*], Jin-Song Shi[2,3], Aidong Yang[1*]

[1]Department of Engineering Science, University of Oxford, OX1 3PJ, United Kingdom

[2]Key Laboratory of Carbohydrate Chemistry and Biotechnology, Ministry of Education, School of Life Sciences and Health Engineering, Jiangnan University, Wuxi, 214122, PR China

[3]Yixing Institute of Food and Biotechnology Co., Ltd, Yixing, 214200, PR China

[4]Oxford e-Research Centre, Department of Engineering Science, University of Oxford, 7 Keble Road, Oxford, United Kingdom

‡Equal contribution

*Corresponding author: jinsonggong.bio@hotmail.com (J. Gong), aidong.yang@eng.ox.ac.uk (A. Yang)

1. Supplementary Methods

1.1 Software and code availability

All scripts were written in python. The deep learning model was implemented using PyTorch v1.12.0. The computer used in this work was a Dell Latitude Laptop with intel core i7 CPU. The model was trained with GPU RTX8000 provided by Advanced Research Computing (ARC) service in the University of Oxford [1]. Figures in the main text and supplementary information were all edited using InkScape (https://inkscape.org/).

1.2 Evaluation metrics

To quantitatively assess the prediction accuracy, R2 (Eq. S1), RMSE (Eq. S2), and MAE (Eq. S3) were computed for each test.

$$R^2 = \frac{\sum_{i=1}^{n}(y_{ie} - y_{ip})^2}{\sum_{i=1}^{n}(y_{ie} - \bar{y})^2} \quad (Eq. S1)$$

$$RMSE = \sqrt{\frac{1}{n}\sum_{i=1}^{n}(y_{ie} - y_{ip})^2} \quad (Eq. S2)$$

$$MAE = \frac{1}{n}\sum_{i=1}^{n}|y_{ie} - y_{ip}| \quad (Eq. S3)$$

2. Supplementary Figures

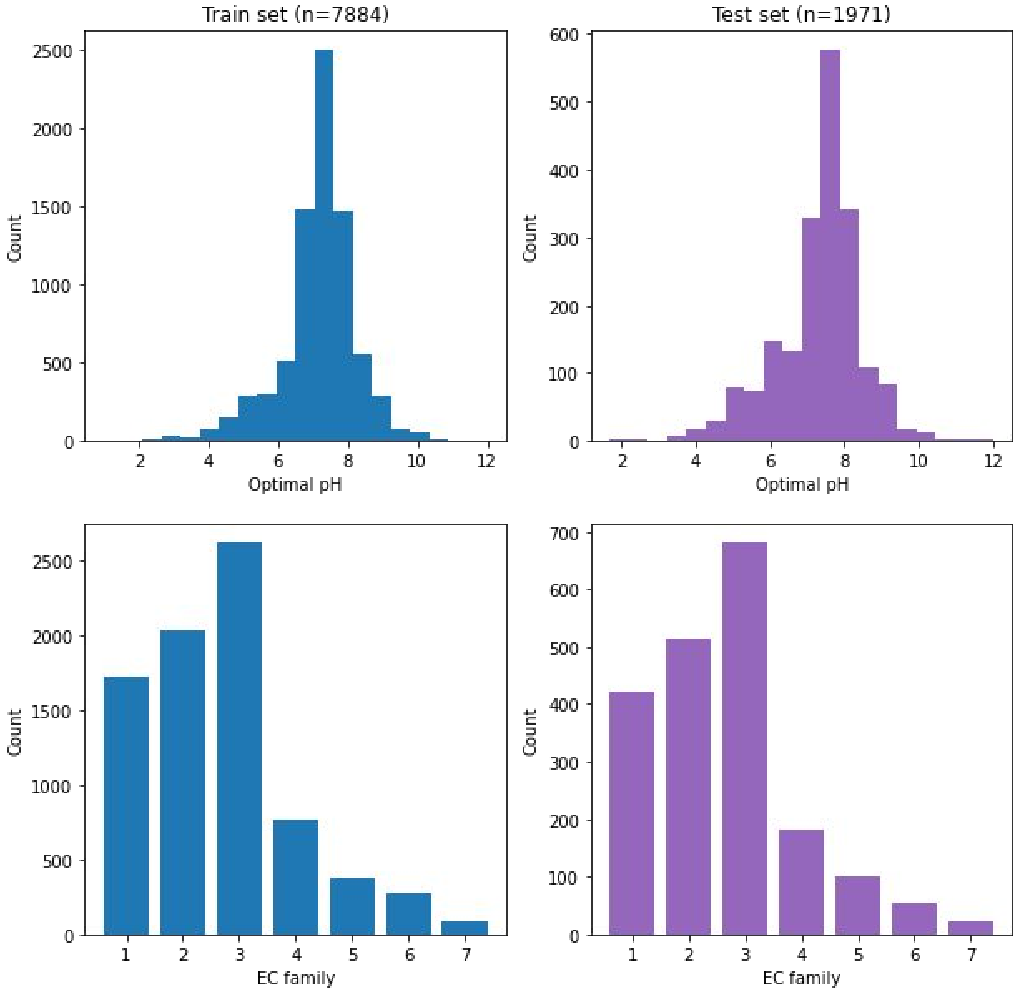

Figure S1. Distributions of enzyme optimal pH values in the training set (7884 entries) and test set (1971 entries), and distributions of enzymes from different EC families in both sets.

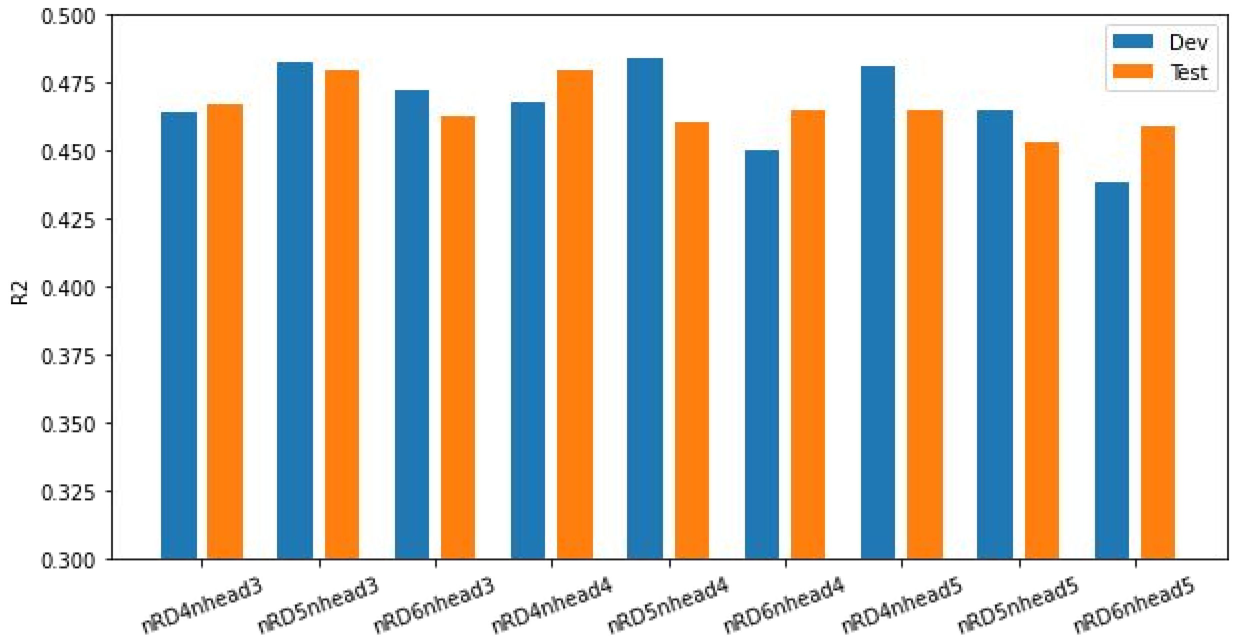

Figure S2. Hyperparameter optimization of enzyme optimal pH prediction for number of attention heads (n_head) and residual dense blocks (n_RD). n_RD=4, n_head=4 was chosen as the best set of hyperparameters.

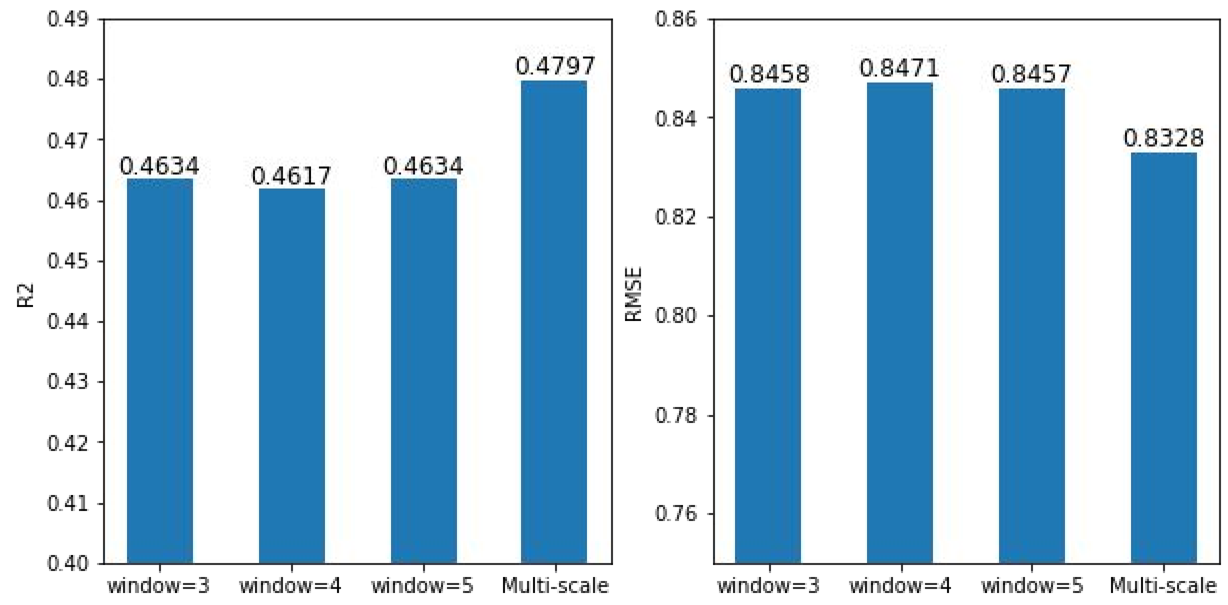

Figure S3. Performance comparison of CNNs with fixed sliding window sizes (3, 4, 5) and multi-scale CNN used in CatOpt. n_RD=4, n_head=4.

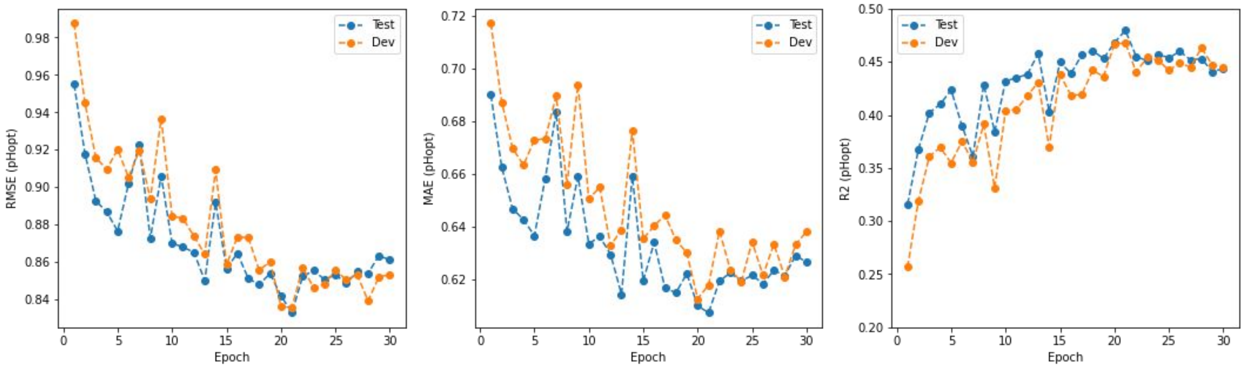

Figure S4. The RMSE, MAE, R2 scores of CatOpt during the training process.

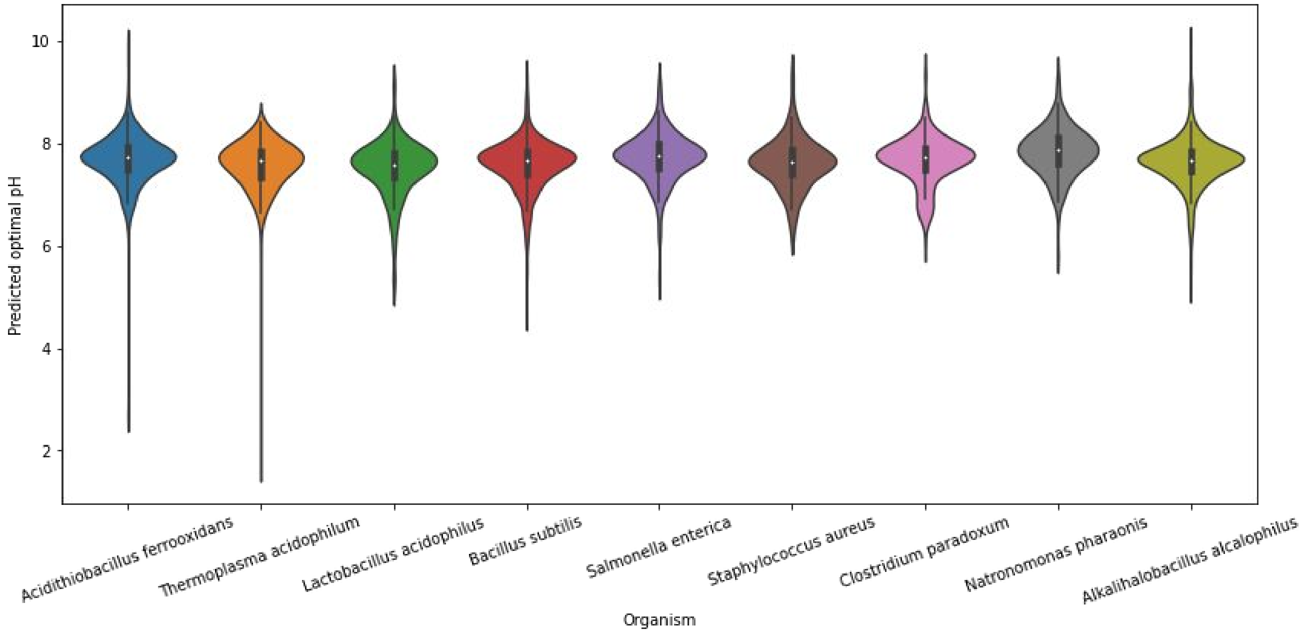

Figure S5. Predicted optimal pH values for enzymes in 9 microorganisms. Acidophiles: *Acidithiobacillus ferrooxidans*, *Lactobacillus acidophilus*, and *Thermoplasma acidophilum*; Neutrophiles: *Bacillus subtilis*, *Salmonella enterica*, and *Staphylococcus aureus*; Alkaliphiles: *Clostridium paradoxum*, *Alkalihalobacillus alcalophilus*, and *Natronomonas pharaonis*.

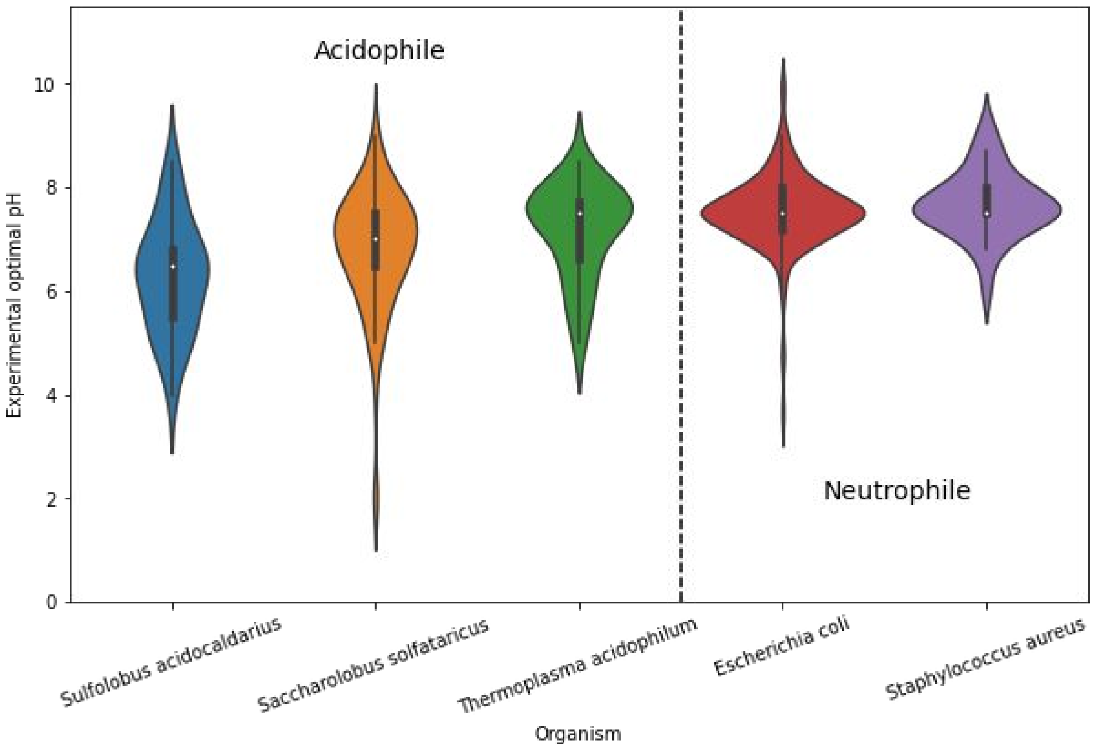

Figure S6. Experimental optimal pH values for enzymes in 3 acidophilic microorganisms and 2 neutrophilic microorganisms.

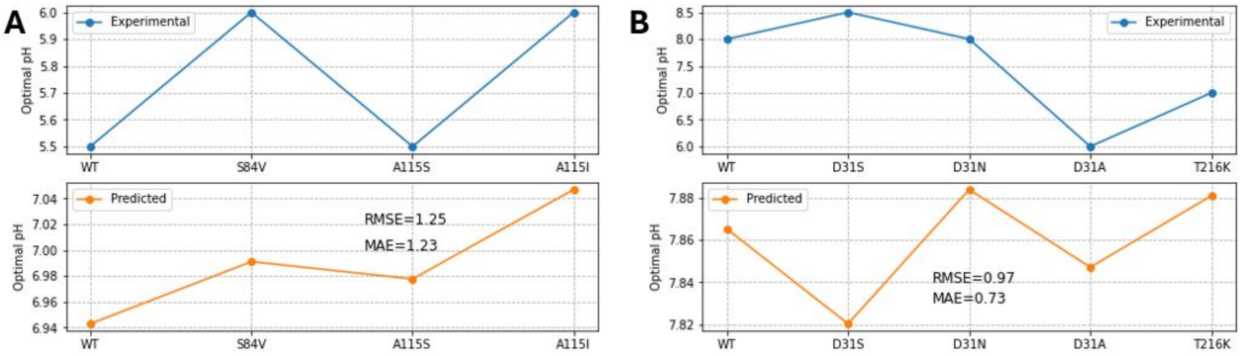

Figure S7. (A) Experimental and predicted enzyme pH_{opt} of wild-types and mutants of BCX. (B) Experimental and predicted enzyme pH_{opt} of wild-types and mutants of CRZHD.

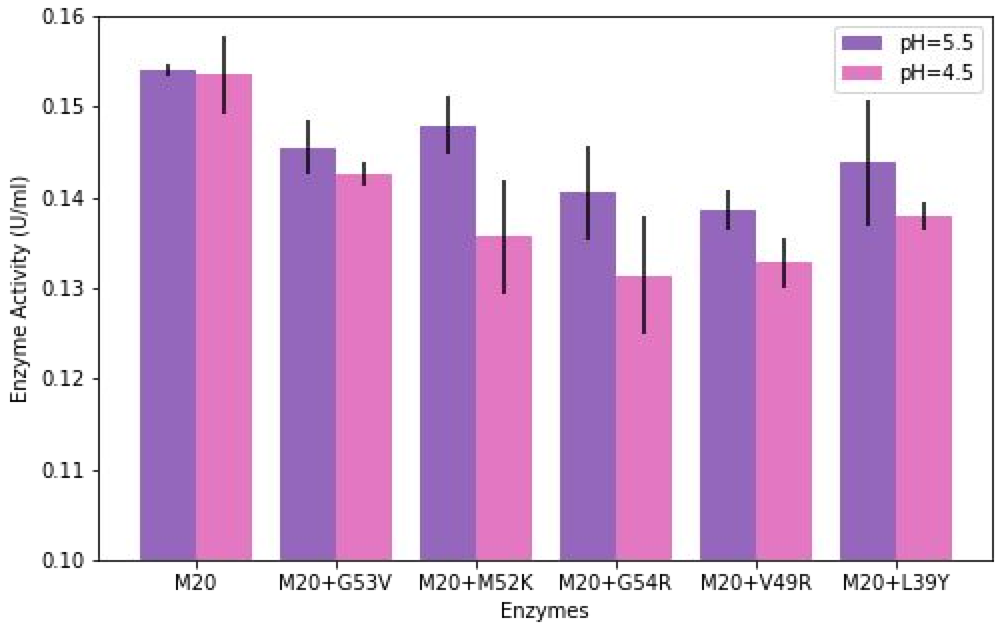

Figure S8. Enzyme activities (U/ml) at pH=4.5 and 5.5 for M20 and M20+G53V, M20+M52K, M20+G54R, M20+V49R, M20+L39Y.

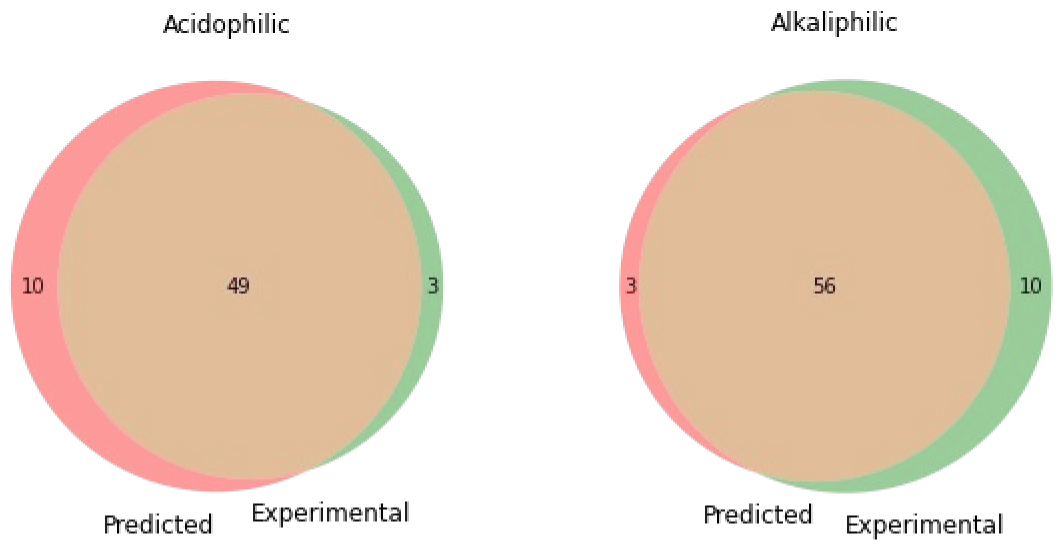

Figure S9. The performance of EpHod on the pH preference of enzymes in the AcalPred dataset.

3. Supplementary Tables

Table S1. Information of wild-type and mutated enzymes used in case studies.

Enzyme	Mutations	pH_{opt}	Reference
Pyrococcus horikoshii diacetylchitobiose deacetylase	M14: G74D/H152E/W232A	7.5	[2]
	M15: G74D/H152E/W232A/Q29E	6.5	
	M16: G74D/H152E/W232A/Q29E/K106E	6.5	
	M17: G74D/H152E/W232A/Q29E/K106E/N176E	6.0	
	M19: G74D/H152E/W232A/Q29E/K106E/N176E/R221E	6.0	
	M20: G74D/H152E/W232A/Q29E/K106E/N176E/R221E/L271E	6.0	
Bacillus circulans xylanase	WT	5.5	[3]
	S84V	6	
	A115S	5.5	
	A115I	6	
Clonostachys rosea zearalenone hydrolase	WT	8	[4]
	D31S	8.5	
	D31N	8	
	D31A	6	
	T216K	7	
	WT	8	[5]

Lactiplantibacillus plantarum tannase	A164S	6	
	F243A	6	
	G77A	8	
	N94A	9	

Table S2. The measured enzyme activities (U/ml) of PhDac mutants at pH=5.5.

Mutant ID	Replicate 1	Replicate 2	Replicate 3
M20	0.1543	0.1532	0.1544
M20+H44C	0.1649	0.1658	0.1672
M20+H44D	0.1454	0.1435	0.1354
M20+H44I	0.1508	0.1504	0.1482
M20+H44N	0.1484	0.1488	0.1487
M20+H44P	0.1456	0.1468	0.1472
M20+G53V	0.1455	0.1484	0.1426
M20+M52K	0.1492	0.1443	0.1503
M20+G54R	0.1369	0.1381	0.1465
M20+V49R	0.1404	0.1390	0.1362
M20+L39Y	0.1514	0.1423	0.1378

Table S3. The measured enzyme activities (U/ml) of PhDac mutants at pH=4.5.

Mutant ID	Replicate 1	Replicate 2	Replicate 3
M20	0.1517	0.1505	0.1585
M20+H44C	0.1629	0.1665	0.1632
M20+H44D	0.1552	0.1558	0.1608
M20+H44I	0.1516	0.1506	0.1524

M20+H44N	0.1534	0.1526	0.1553
M20+H44P	0.1494	0.1523	0.1508
M20+G53V	0.1426	0.1440	0.1412
M20+M52K	0.1328	0.1313	0.1429
M20+G54R	0.1355	0.1239	0.1347
M20+V49R	0.1306	0.1319	0.1358
M20+L39Y	0.1393	0.1381	0.1364

References

[1] A. Richards, University of Oxford Advanced Research Computing, (2015). https://doi.org/10.5281/zenodo.22558.

[2] Z. Huang, X. Mao, X. Lv, G. Sun, H. Zhang, W. Lu, Y. Liu, J. Li, G. Du, L. Liu, Engineering diacetylchitobiose deacetylase from Pyrococcus horikoshii towards an efficient glucosamine production, Bioresour. Technol. 334 (2021) 125241.

[3] S.H. Kim, S. Pokhrel, Y.J. Yoo, Mutation of non-conserved amino acids surrounding catalytic site to shift pH optimum of Bacillus circulans xylanase, J. Mol. Catal. B Enzym. 55 (2008) 130–136.

[4] A. Dotsenko, I. Sinelnikov, I. Zorov, Y. Denisenko, A. Rozhkova, L. Shcherbakova, The protein engineering of zearalenone hydrolase results in a shift in the pH optimum of the relative activity of the enzyme, Toxins (Basel) 16 (2024). https://doi.org/10.3390/toxins16120540.

[5] H. Pan, J. Zhan, H. Yang, C. Wang, H. Liu, H. Zhou, H. Zhou, X. Lu, X. Su, Y. Tian, Improving the acid resistance of tannase TanBLp (AB379685) from Lactobacillus plantarum ATCC14917T by site-specific Mutagenesis, Indian J. Microbiol. 62 (2022) 96–102.
